# 155. Use of MRSA PCR and Antimicrobial Stewardship Intervention to Limit Anti-MRSA Therapy in Respiratory Tract Infections at a Community Teaching Hospital

**DOI:** 10.1093/ofid/ofad500.228

**Published:** 2023-11-27

**Authors:** Paula A Politis, Alice Chen, Michael J Oravec, Matthew England, Michael Tan, Thomas M File

**Affiliations:** Summa Health System, Akron, Ohio; Summa Health, Akron, Ohio; Summa Health System, Akron, Ohio; Summa Health, Akron, Ohio; Summa Health System, Akron, Ohio; Summa Health System, Akron, Ohio

## Abstract

**Background:**

Patients admitted to the hospital with respiratory tract infections (RTIs) who are at risk for MRSA are often treated empirically with anti-MRSA agents such as vancomycin or linezolid. While pre-treatment respiratory culture is recommended, it is not always feasible. An alternate, more easily obtainable test is the MRSA nasal PCR, which is used to detect nasal colonization. While a positive nasal MRSA PCR cannot determine MRSA pneumonia, a negative MRSA PCR result strongly correlates with lack of MRSA pneumonia providing a useful antimicrobial stewardship tool for de-escalation or avoidance of empiric anti-MRSA therapy. Our Antimicrobial Stewardship Program (ASP) routinely follows patients admitted with RTIs and provides recommendations for appropriate therapy based on culture and test results, including the MRSA PCR. The purpose of our quality improvement project was to assess the practice and clinical impact of reduction of specific MRSA therapy associated with a negative nasal MRSA PCR.

**Methods:**

We performed a retrospective review of patients admitted to our institution with MRSA PCRs performed for the purpose of RTI workup from November-December 2022. We compared patients with negative MRSA PCRs (not detected or MSSA) with and without ASP intervention and assessed differences in patient characteristics and outcomes. If ASP intervention occurred, intervention and acceptance data was collected. Characteristics were compared using descriptive statistics.

**Results:**

Shown in Table 1 and 2. There was no significant difference in age, sex, duration of MRSA therapy, SCr, length of stay, or readmission between groups. When looking at ASP intervention for 1. discontinuation of MRSA therapy or 2. ordering/collecting MRSA PCR, a total of 73 interventions occurred with a 100% acceptance rate. This encompassed 34% (52/155) of patients on MRSA therapy with a negative PCR result.

**Figure:**
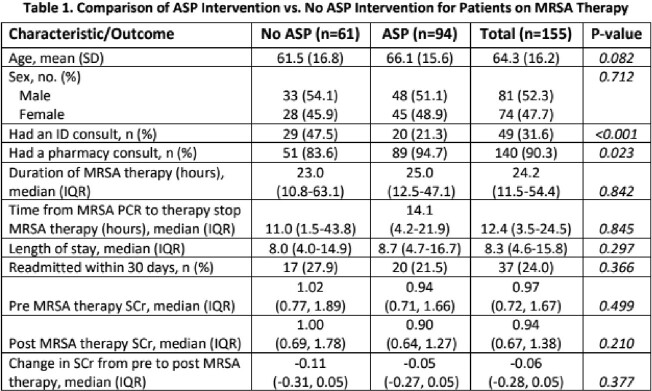

**Figure:**
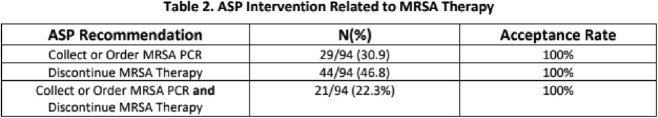

**Conclusion:**

While no significant difference was found between groups in terms of duration of MRSA therapy, the slightly shorter duration in the non-ASP group was likely due to prior discontinuation of MRSA therapy thus not necessitating ASP intervention. Those with slightly longer duration received ASP intervention which led to discontinuation of unnecessary MRSA therapy.

**Disclosures:**

**Thomas M. File, Jr., MD, MSc, MACP, FIDSA**, HealthTrackRx: Advisor/Consultant|NAbriva Therapeutics: Advisor/Consultant|ThermoFisher Scientific: Advisor/Consultant

